# The cascade of social determinants in producing chronic disease in low-income African-American men

**DOI:** 10.1080/17482631.2018.1549920

**Published:** 2018-12-12

**Authors:** David Buchanan, Aline Gubrium, Lamont Scott, Henry Douglas

**Affiliations:** a Department of Health Promotion & Policy, School of Public Health & Health Sciences, University of Massachusetts, Amherst, MA, USA; b Men of Color Health Awareness, Springfield, MA, USA

**Keywords:** Health disparities, African-American, men’s health, cultural competency

## Abstract

**Purpose**: There is a dearth of effective, evidence-based programs to reduce chronic disease in low-income African-American men. We report on the results of formative research in the National Institutes of Health (NIH)-funded MOCHA Moving Forward project on factors identified by the participants to drive health disparities.

**Methods**: Based on individual interviews with 42 middle-aged (40–65 years), low-income African-American men, three themes emerged.

**Results**: First, the results indicate a hierarchy in the perceived relative influence of different factors, with poverty and unemployment perceived to have the most powerful affects. Second, results show that factors in different domains do not operate as discrete independent influences, but rather, interact synergistically. Finally, the findings show how perceived social structural constraints have produced deep cynicism about the future, with notably divergent reactions, producing a sense that there is almost nothing an individual can do, or paradoxically, a greater the sense of personal responsibility.

**Conclusion**: The implications of addressing the cascade of social determinants to reduce chronic disease in African-American men are discussed.

## Introduction

The first official national call to reduce health disparities in the USA was issued in the 1990 Surgeon General’s Report, *Healthy People 2000*, and reiterated in *Healthy People 2010* and *Healthy People 2020*. Despite the high priority placed on this national goal, progress over the last 30 years has been slow, and to the extent that progress is being made, health disparities are becoming further concentrated in poorer populations with lower levels of education. (Ding, Do, Schmidt, & Bauman, 2015; National Center for Health Statistics, 2017; Taylor, Dal Grande, Wu, Shi, & Campostrini, 2014) Thus, re-doubled efforts to develop innovative programs tailored to the needs of historically underserved populations are critical.

The Men of Color Health Awareness (MOCHA) program is a grassroots, community-driven movement to improve the health of low-income African-American men who bear a disproportionate share of the burden of health inequities. Started in 2012 in a large New England city, MOCHA runs a structured 12-week program that combines small group discussions on issues facing men of colour once a week with aerobic exercise classes two days a week, in addition to conducting community outreach education and other activities. University researchers were asked to document the impact of MOCHA in 2014, and were awarded National Institutes of Health (NIH) funding to conduct a randomized controlled trial on its effects. The first 18 months of the *MOCHA Moving Forward* study have focused on conducting formative research to identify the most salient risk factors influencing African-American men’s health. The key research question driving the analysis presented here was “Which factors do low-income, middle-aged African-American men perceive to have the greatest impact on driving health disparities?” The formative research seeks to identify key mediating variables so that they can be operationalized and empirically measured in the experimental research phase and/or incorporated into future versions of the MOCHA model. This paper reports on initial findings of the formative research.

The purposes of this paper are: (1) to describe and categorize participants’ perceptions of the key risk factors influencing African- American men’s health; (2) to illustrate how the identified specific factors interact across domains; and (3) to formulate the major emergent themes that best capture the results of the analysis in summary fashion. As demonstrated by the results of this research, the hierarchical ranking of the relative influence of different factors, specific information about the direction and types of interactions across domains, and the paradoxical reactions to perceived social constraints, whether to give up caring about one’s personal health or to assume greater individual responsibility, provide important new information about the complexity of factors influencing African-Americans health, with strong implications for the development of more effective interventions.

## Need for effective programs

For readers who may be less familiar with health and social indicators of African-Americans living in the USA, African-American men die on average 5.1 years sooner than non-Hispanic Caucasian men (69.6 vs.75.7 years) and they face higher rates of illness and mortality. (Barr, 2014) The rates at which African-Americans experience fair or poor health is nearly twice as high as that of non-Hispanic Caucasians. While 27% of non-Hispanic Caucasians ages 35–64 years living in the USA have hypertension, 43% of African-Americans experience hypertension. Nearly 38% of non-Hispanic Caucasians adults are obese, compared to 46.8% of African-Americans. African-American men have higher rates of heart disease than White men (44% vs. 37%, respectively), while the prevalence of diabetes is 13.4 (per 100,000) for African-Americans vs. 7.3 for non-Hispanic Caucasians, almost twice as high. Approximately 75% of African-American men and women develop high blood pressure by age 55 compared to 55% of non-Hispanic Caucasian men and 40% of non-Hispanic Caucasian women of the same age.

Compared to non-Hispanic Caucasians, African-Americans face the same risk of unemployment today as in the 1960s. Between 2007 and 2013, the net wealth of the median African-American household fell from 10% to 8% of median non-Hispanic Caucasian household wealth. The median non-Hispanic Caucasian household now has a net wealth 13 times greater than the median African-American household. In 2000 the median African-American household had an income that was 66% of the median non-Hispanic Caucasian household income; in 2015 that figure was 59%. Fully 33% of African-American children under the age of 16 are growing up in poverty, compared to 10% for non-Hispanic Caucasian children. (National Center for Children in Poverty, 2018)

There is a paucity of strong evidence-based interventions that have been demonstrated to reduce chronic disease disparities among low- income African-American men with low levels of education. (Centers for Population Health and Health Disparities [CPHHD], 2007) The dearth of effective programs remains true despite frequent calls over the past several decades for developing “culturally competent” programming. (Betancourt, Green, Carrillo, & Ananeh-Firempong, 2003; Betancourt, Green, Carrillo, & Park, 2005; Institute of Medicine, 2002, Watt, Abbott, & Reath, 2016) One reason for the apparent gap is the inherent tension between the scientific value attached to the principle of generalizability and the perceived need to modify programs to address local needs, the now familiar fidelity/adaptation dilemma (Buchanan, 2015) (a problem being investigated in initiatives such as the CDC’s Diffusion of Evidence-Based Interventions (DEBI) project; see https://effectiveinterventions.cdc.gov/). For our purposes here, it suffices to say that, while programs such as the Diabetes Prevention Project have demonstrated reliable success in the general population, (Diabetes Prevention Program Research Group, 2002) greater benefits accrue to the majority segment of non-Hispanic Caucasian, middle class, well-educated persons than other population groups. (Gwatkin, Bhuiya, & Victora, 2004) The initial hypothesis guiding this research is that the constellation of factors and how they operate to affect chronic health conditions in low-income African-American men are distinct from those affecting other population groups. Thus, the need for conducting further exploratory research.

## Research methods

MOCHA Moving Forward is designed as a Community-Based Participatory Research investigation. (Buchanan et al., 2007; Israel, Schulz, Parker, & Becker, 1998; Minkler & Wallerstein, 2003) This approach was adopted for both ethical reasons pertaining to respect for community autonomy, and scientifically, to minimize latent assumptions and/or potential social desirability biases in data collection and analysis. Because the research was designed to examine the causes of “problems” or “pathologies” in the African-American community, we thought it ethically imperative to work with the community as closely as possible to minimize any potential misunderstandings or misrepresentations of the social dynamics contributing to health disparities, and thus, to the design of interventions to “fix” the problems, critical ethical issues raised by Shelby (2017) that we return to in our concluding remarks. The two lead investigators spent two years attending weekly meetings of the MOCHA Steering Committee prior to the award of NIH funding. This period was critical to build trust and a common understanding of the purposes of the collaboration. During this time, we discussed the fact that MOCHA had grown up organically as a result of outcry against persistent health disparities in the community, while the research would offer an opportunity to evaluate program effects objectively. For example, where the MOCHA program implicitly recognizes the influence of gender role stereotypes on African-American men’s health behaviours (addressed in the MOCHA curriculum in the “Unpacking the Man Box” session), it is important to capture the hypothesized effects in an empirical scientific framework.

During the formative research phase, although we collected data using three formative research methods—oral histories, digital storytelling and semi-structured health interviews—here we report only on the analysis of the semi-structured individual interviews. A three-member research sub-committee of the MOCHA Steering Committee was formed to collaborate with university researchers on developing the health interview protocol. Over the course of three meetings, the research team agreed on a set of five open-ended questions intentionally designed to be non-directive with respect to the participants’ perceptions of the causes of health disparities and recommendations for addressing them (see Table I). For the health interviews, we sought to recruit 40 African-American men aged 40–65 years, purposively selected to include 20 men in good health and 20 men experiencing chronic disease conditions, defined as diabetes, hypertension and/or obesity. The projected sample size estimate was driven by considerations of a minimally adequate sample to enable meaningful comparisons across health status, while keeping open the option of conducting additional interviews until saturation of identified factors was achieved. (Marshall, 1996; Miles, Huberman, & Saldana, 2013; Sandelowski, 1995) The research sample was recruited by MOCHA mentors using the same methods that the MOCHA uses to recruit its program participants, (Graham et al., 2018) broadly, through street outreach, outreach at targeted community events, and the strong social networks of the MOCHA men; thus, it is representative of men who participate in the MOCHA program.

**Table I.** MOCHA interview protocol.

1. Tell me about what you learned while growing up about why some people get sick, while others stay healthy.
2. What does “being healthy” mean to you? What are some things that you think help people to be healthy or feel good about themselves?
3. What are some things that you think make it hard for people to be healthy or feel good about themselves? Was there anything in the last couple of weeks that you did that was probably not so good for your health?
4. Many people talk about health disparities these days. Why do you think that African-Americans have higher mortality rates than other races or ethnicities? What do you think causes these disparities?
5. What do you think we need to do to change, reduce, or eliminate health disparities?

The interview protocol and Informed Consent procedures were reviewed and approved by the **University of Massachusetts, Amherst,** Institutional Review Board prior to initiating data collection *(approval number 2016–3257)* to assure conformity with US Federal Law (45 CFR 46, Protection of Human Subjects) and the ethical principles on health research with human beings set forth in the World Medical Association’s Declaration of Helsinki. The IRB determined that the research protocol fulfilled all of the requirements for research with human subjects, including the provision of information on the purpose and procedures of the study, procedures for protecting confidentiality, an assessment of potential risks and the safety of the participants, the right to withdraw from the study at any time without penalty, and that the research was guided by the ethical principles of autonomy, beneficence, non-maleficence and justice. The process of obtaining informed consent was conducted in-person, one-on-one both orally and with written documentation, with ample opportunity to address any questions or concerns.

In addition to upholding the rights of the participants and providing protections against exposures to any unnecessary or undue harms, as researchers, we were concerned a priori *about the possibility that the results might reproduce and reinforce negative social stereotypes that could be due to internalized racism, whereby the interviewees might attribute unhealthy behaviours to individual character flaws. We decided that we wanted the analysis to stick as closely as possible to the actual language used by the participants, as such information would provide a baseline of perceptions of a key factor that we hypothesize the intervention is designed to change and because there are open unresolved questions about the degree to which individuals can make autonomous decisions about their behaviours despite social structural pressures and constraints. To guard against perpetuating possibly offensive images or portrayals, we used a more extensive process for vetting the analysis with community members than is used in traditional scientific study designs, presenting our preliminary analyzes to the MOCHA Steering Committee and circulating drafts to independent African-American scholars requesting their feedback and endorsement before submitting for publication.*

Consistent with CBPR principles, MOCHA Steering Committee members, all African-American men with deep ties in the community, both recruited potential participants and conducted the individual interviews. The final sample size for the individual interviews was 42, with an average age of 49.4 years. Here we report on the results of the overall analysis; the results of comparative analyzes of healthy and unhealthy men are forthcoming.

Although CBPR is widely advocated as an important means for uncovering new insights into stubborn health problems, the candour elicited by these homophilous conversations is notable. As one respondent said,

If a white guy was asking me these questions, I would be feeling a little like, “Why would I share this with you?” You see what I’m saying? It’s hard talking to somebody who’s not in my culture. That’s another thing. I might not feel comfortable to open up. I might not feel comfortable opening to [a white person], because he’s not gonna understand me. I’m not trying to offend—I’m not saying nothing; I’m just saying that’s what I’m talking about. There’s only certain people you can talk to. There’s certain people you’ve got to actually open up, so they can break through that, so that the next time, I can talk to him [white person]. But right now, that’s not going to happen.

Similarly, another participant commented on the inherent power/status differential in feeling patronized by university researchers:

They [community members] feel like… it’s hard. I think they feel like they think they have to prove themselves to somebody else. They feel ashamed, embarrassed. They feel like they’re babying them.

We used a recursive process to analyze the interviews. Typically, the MOCHA interviewers completed 2–3 interviews each week. After the interviews were transcribed, the three MOCHA research team members and one faculty member ([authors removed for blind review]) met once a week to review the transcripts, during an open coding stage. Discussions were wide-ranging, from the specifics about what a particular respondent meant by a particular phrase (e.g., “kicking the bobo”), to reflections on the “big picture” challenges facing the community; thus, we moved back and forth between the details stated in individual responses to the broader context of life in contemporary American society, an iterative method essential for situating and interpreting the participants’ observations. (Miles et al., 2013) As new codes and themes emerged, we re-examined previously discussed transcripts to look for similar themes, and nuances in variations on a theme. Graduate student research assistants helped codify, systematize and re-code the interview transcripts with a draft working coding manual using NVivo 11; initial raw codes were categorized into different nodes and the tree node function was used to identify interactions between nodes.

To corroborate the credibility of emergent themes, (Denzin & Lincoln, 2000; Patton, 2001) we sought out member-checking feedback from the MOCHA Steering Committee, and we organized a working lunch where the preliminary results were presented to a group of eight interview participants. The following reflects a working consensus at this point in the research.

## Results

In overview, factors identified in the interview protocols were categorized into four domains, ranging from social structural influences to individual effects. Within this framework, three overarching themes emerged in the analysis. First, the results illustrate a clear hierarchy in the perceived relative influence of the different factors identified by the respondents, with poverty and unemployment perceived to have the most powerful affects. Second, factors identified in different domains do not operate as discrete independent influences, but rather, they interact synergistically, magnifying one another over and above any simple linear additive effect. The results show a cascade (ripple or snowball) effect of issues in one domain generating new problems in another domain. Third, the findings show how perceived rigid social structural constraints have produced deep cynicism about prospects for a better future. Notably, the results reveal divergent reactions to perceived social structural constraints, producing either a sense that there is almost nothing an individual can do, or paradoxically, a greater sense of personal responsibility for health.

In presenting the results, we start at the individual level, with the participants’ perceptions of the holistic nature of health. We start here to highlight the strong mind-body connection expressed by the participants, and to draw attention to the contrast between the respondents’ views and the emphasis placed on physical factors—diet, exercise, non-smoking—in most public health programs designed to reduce chronic disease prevalence. We then step back to examine the level of social structural factors, highlighting the interaction between unemployment and racism in a market economy. Next, we move to institutional factors, such as housing, social services, and transportation, and provide examples to illustrate the dynamic interactions across domains. We then turn to influences at the interpersonal level, and finally, close with a more detailed examination of individual factors.

### Individual perceptions of health

The interviews began with a friendly “ice-breaker” question about what the participant had learned about staying healthy while growing up, and then asked, “What does being healthy mean to you?” In the analysis, the view that health is something that goes beyond diet and exercise but rather is seen in terms of a holistic, integrated mind-body phenomenon emerged as a consistent theme. Two examples capture the most common view about what being healthy means to the participants:

Keep my mind in a stable right state of mind. It’s all in the mind, in your heart, and in your spirit—what you feel like you want to do.

First of all, healthy means to me is working on my self-esteem—my state of mind. I’m as positive as my mental psyche. That’s… which is basically I have anxiety. I allow these stressful things to affect me. As it affects me, it’s going to affect my health. It’s going to affect me mentally, spiritually, and physically, right, and I realize that.

The phrase, “physically, mentally, and spiritually,” came up repeatedly in participants’ discussions of what health means to them.

### Social structural influences

When asked about the causes of health disparities, participants identified a small set of key influences, which map well onto various lists of the social determinants of health. (Marmot, 2005; Wilkinson & Marmot, 2003) The overwhelming majority saw that unemployment and poverty—which are significantly aggravated by racism in the competition for jobs—drive the majority of problems that they experience in different spheres of daily life.

#### Unemployment and poverty

Participants repeatedly emphasized that the issue that makes it most difficult for them to stay healthy is the lack of jobs. Virtually all of the problems faced by poor minority communities were traced to the lack of steady employment and stable income.

First of all, if I don’t got no money, I’m stressed, I’m miserable, I’m unhappy, there’s nothing to talk about. If you’re broke and you can’t even afford to—if you’ve got a girl, you can’t take her to a movie or out to eat, you can’t go buy yourself no shoes, no sneakers, no clothes. You don’t have a job, you don’t have insurance, you can’t even go to the doctor. This is not good.

Bills—not being able to pay your bills. You can’t pay your rent. You can’t pay your gas or your light bill. That’s going to stress you out.

One man succinctly captured the principal challenge in reducing health disparities: “When we come out of the womb, we’re already stuck into a piece of poverty.”

#### Racism

Closely associated with problems of poverty and unemployment, participants noted the persistent effects of racism. While racism has been diagnosed at institutional, social and individual levels, (Jones, 2000) most of the participants saw racism as a systemic, societal state of affairs, rather than individual acts of bigotry, as the following examples illustrate:

It also becomes a pressure for us to live in a society that doesn’t want us to—that wants to continue to keep us down.

For an African-American man, this here system is not set up for us anyway. Of course, that’s going to stress you out.

We’re adapting to an environment that really is not ours, but we have to adapt to that just to survive. That’s going to create a tremendous amount of stress.

It’s horrible. Sometimes I sit in my room and I’m like, “Wow,” and I think about how we grew up. When I was in North Carolina, my family used to say, “You got to walk on this side of the street because the white people on this side and you can’t be on the same side as them.” But I had the heads-up, so I knew what to expect. Today, racism is so embedded and so sneaky and so snake-wise that you just don’t know. It’s more sneaky now because, see, back then, you knew what you was looking at, you knew that the “massa” didn’t like you, you knew that this don’t like you. Now, you got people who act like they like you, and then they’re doing stuff to destroy you, and a smile on their face. It’s a different way now. Now, it’s crazy because you don’t know who to trust or which way to turn.

At the time of these interviews, there had been a resurgence of killings of young African-American men by police in the USA. This oft-mentioned aspect of racism will be addressed in a separate paper.

#### Interaction of unemployment and racism

Although participants tended to see unemployment as the primary problem they face, they noted that racism consistently tipped the scales against them in the competition for jobs in a market economy.

I think maybe in an urban environment, the stress of a black person may be a little higher than a different demographic for competitive reasons when it comes to jobs and things of that nature… My stress levels are just high to be able to survive.

Yeah, the economic thing, the job thing, and just some racism thing. There’s a little bit of everything, man, that’s keeping us at a disadvantage. We know that we’re at a disadvantage, and so we’re under more stress to succeed, to find work.

Notably, the men were aware that the need for work often pitted them against one another, “We’re not helping one another out, because we are too busy pulling each other down because we have been like crabs with one another.”

### Institutional level

In descriptions of the Social Determinants of Health, (Marmot, 2005; Wilkinson & Marmot, 2003) poverty and unemployment are typically listed on par with other social determinants, like education, transportation or housing. Participants in this study, however, described a clear hierarchy of influences. Our results indicate that unemployment and poverty, exacerbated by racism, are the main driving forces generating problems in other spheres of life.

In examining the impact of poverty, unemployment and racism, the results of this study show how a problem in one domain cascades, ripples or snowballs to create problems in other spheres. For heuristic purposes, the different spheres of life can be categorized by their relation to primary needs and basic goods, such as needs for food, housing, and shelter. The respondents described problems that they experience in meeting basic needs in many different areas; due to space constraints, the results presented here are limited to the five most common concerns: looking for work, housing, neighbourhood infrastructure, transportation, and diet/access to healthy food.

#### Looking for work

The need to find decent work was foremost in the participants’ minds with respect to the day-to-day challenges facing their community. The toil and toll of looking for work and fear of losing one’s job came up frequently:

I would say, trying to find a job. That might be stressing them out…. It’s not *what* you know, you know, it’s *who* you know, that kind of thing.

Right now, the thing that’s on my mind is, the company that I’m working at right now, we’re going through a merger right now. When they have mergers, the company that comes in, they might do a lot of cutting. It sounds good—we’ve got to do this. Right now, I’m just still wondering if my job is on the line right now. That’s been stressing me.

#### Housing

After employment, the most common source of stress related to the effects of poverty and racism on housing, with two distinct aspects: segregation and homelessness. The participants saw that housing and rental properties are strongly segregated by neighbourhood, and the threat of eviction loomed over many men’s heads. They perceived little choice but to live in neighbourhoods with high poverty rates:

The housing as well, places to live, and to me, we are discriminated against. That’s how I feel.

That’s the same in our poverty and what I like to call a disinvested area, because it’s an area they don’t put money into, which is commonly known as high-poverty areas.

One man noted the way that African-American residents are segregated into certain neighbourhoods where drug use is possible yet contained there:

We can use because of the place we live—our surroundings, the way they put us in certain areas and certain places within the setup directly in a drug or high drug area, right.

Even in poor neighbourhoods, however, meeting monthly rent payments was a constant struggle and many had experienced or witnessed the trauma of being evicted and periods of homelessness:

My situation took a turn for the worse, but it was pretty bad when I ended up being homeless because of the landlord. She was money hungry, so the city found out, I got caught up in the whole mess, and even though I was trying to move forward, I got shot down. The bad thing was the stress, the stress that I was thrown into being homeless again.

#### Neighborhood infrastructure

Closely related to housing segregation, participants perceived that the level of public services varied markedly by neighbourhood:

[We’ve got to] Go to the government and let them know the roads need fixing. Let us have something decent in the neighborhood…. You go to, you go out to [name removed], you go out to [name removed], you find out in them neighborhoods got some place for their people—why can’t we have some place for our people in the city?

I asked the nurse about some x-rays because I needed an x-ray done. She said, “Our machine is down.” I asked, “How long has it been down.” She said, “A couple of weeks.” I said, “Why?” She said she didn’t know, but they were going to come next week to get it fixed because there were patients that needed it. I didn’t believe her and I told her, “We’re in the ’hood, so we get ’hood services. We’re not getting that machine fixed anytime soon.”

#### Transportation

Another major obstacle cited was the lack of adequate transportation. Difficulties in getting around were frequently mentioned as a significant barrier in meeting basic needs:

When you don’t have a car and when you’ve got to be able to depend on somebody else to give you a ride from point A to point B, that’s stressful.

Don’t have transportation—can’t get there—don’t have the money to get there.

Black people that got cars, when you drive down the street, your car is breaking down.

#### Food access

Finally, virtually all participants noted the relationship between income and diet, specifically, how they had to resort to consuming cheap food because they could not afford any better. In their view, one has to figure out what to eat on a “broke budget”:

Yeah, because I was a project kid, so I know what it’s like to eat beans with white rice three to four days a week, a bread-and-butter sandwich a couple of days a week. You can survive off it. It doesn’t mean that it’s meant to be that way, that that is nutritious.

Sometimes they don’t have the money they can afford these healthy foods, so they just go to the cheapest aisle. For a long time, that’s what I was doing, see what I can make on a broke budget. I was living off of ramen noodles and hotdogs. I tried switching it up, throwing in one of those canned soups, like cream of chicken, cream of broccoli into the ramen noodles, but that’s all I could afford at the time.

### The cascade effect

After the perceived hierarchy of felt affects, a second major finding to emerge in this research is the snowballing of effects across domains, how a problem that originates in one area spills over to generate additional problems in other areas. We present a limited number of examples to make the point within space constraints. The first example illustrates how issues in housing impact diet:

You can’t get that healthy food that you’re talking about. The money is just not there. When it comes to getting healthy food or paying the rent, I think it’s self-explanatory. You make the sacrifice…. when the money gets low, you go to straight up starch.

Similarly, transportation difficulties spill over into problems in accessing healthy foods. One participant noted,

I know for me, transportation, being able to get from [name removed] to a farm in [name removed] to get some organic, fresh produce is not happening anytime soon. I know they’re out there, but it’s just not accessible to me.

Another remarked that it is even difficult to get to the nearest grocery store because of transportation costs:

Part of the reason is that we can’t afford the fresh vegetables. We can’t afford to keep going back and forth every day to the store. Yes, we have farmer’s markets that are there weekly, but if you’re buying fresh vegetables on that Monday, they got to be cooked within the next day or two, and you’re back at the store again. Our population doesn’t have the resources to keep running back and forth to the store to fill up their refrigerator.

The example of the broken x-ray machine cited above illustrates how one’s neighborhood of residence limits accessibility to essential health services. It is also the case that issues in two different domains may combine to disrupt access to basic goods in a third domain:

My situation took a turn for the worst, but it was pretty bad, when I ended up being homeless because of the landlord… The bad thing was the stress, the stress that I was thrown into being homeless again. I was supposed to have surgery, but I couldn’t because I didn’t have anybody to pick me up. I told them, “I’ll be fine. I’ll get a taxi just like I got a taxi to get here.” They told me that’s not going to happen and I couldn’t have my surgery. Because of the situation I’m in right now, I don’t have a place to rest [due to being evicted] for the two weeks that are required after the surgery, so I’m not going to get the surgery. That was months ago. I’m still in pain. Still really need that surgery.

### Interpersonal level

When asked about what helps men stay healthy and the causes of health disparities, a surprising finding was the omission of mentions of the benefits of social support. More often, the men spoke of the difficulties of providing for their family.

When you have a family and you can’t provide for them, you know, that’s stressing.

It’s d**n sure a shame for a parent to not be able to provide a decent meal for their kid.

The strain of not being able to provide for one’s family in turn took its toll on extended family relationships:

I think what really stressed me out is the fact that I don’t have the relationship that I should and I want to have with my family.

To me being healthy means that you’re emotionally stable, being able to hold and maintain healthy relationships with people, not just, not f**k them up, not f**k up the relationships with everybody, family, and people, in general.

The implications of the felt isolation and lack of social support are examined in the discussion section below.

### Individual-level redux

Returning to the individual level, three issues were most frequently brought up in the interviews: the high salience of stress; deep cynicism about the future; and the divergent reactions to this time-hardened pessimism, whether in giving up and not caring about one’s health, or in viewing it narrowly, strictly as a matter of making the right personal choices in diet and exercise, irrespective of other burdens and the limited options available.

#### Stress

Stress was reported as a major, constant factor in these men’s lives. The respondents frequently noted how their mental health affects their physical health.

I think—in my opinion—I think stress plays a part. I do honestly believe that—that African-American—Black men have the highest stress. For whatever reason, it may be—maybe because of our generation—I don’t know, but I’ve seen a lot of people—Black people—African-American men pay a big part of their health by stressing.

You pick up bad habits, and your health starts to deteriorate because of the stresses of life. That’s a constant war. It’s a battle that you go through.

The men used vivid language to describe the experience. “Stress eats away at you. I know it eats away at me.” It “leeches and vampirizes your soul, you feel tired.” It’s “psychological genocide, or what I also call a psychological maze. We’re stuck in a psychological maze, because we don’t know which way to turn… That affects our health, as well, because psychologically we’re just not there.” The high level of stress was also seen to set a vicious cycle in motion:

There’s a certain amount of stress in every day. Negative stress—if you’re stressed out, like a hard day at work and you get home and you have a problem with the family and it escalates, right, from having a problem with the family—a misunderstanding with the family unit. Take that—that goes from there to the bar. Yes, I said the bar. Now you’re with them, with those negative people and you’ve ordered a drink. Right now, your stress has tripled. Now you went from there to something negative and dangerous. [Interviewer: Such as?] Putting yourself in a bad situation. You went from there to—now you’re at a level where you’re drunk and you’re angry, right, and triggers start kicking in. You have attitude. All of a sudden, now can’t nobody approach you. Whoever approaches you, you’ve got your arm up—you’re ready to fight. You’re ready to get busy. This individual may be reaching out to help you. You don’t even realize that because you done went from two shots to a fifth, right? Now you’re just ready to hurt somebody. You’re putting yourself in a negative and dangerous—stressful situation.

The high level of stress was the major critical difference that they saw between the races: “What do they say, white privilege is—they have reason to be stressed, but brothers are more stressed. Definitely. They’re not worried about a lot of things that Black people worry about.”

#### Deep cynicism

Among these middle-aged men, the interviews highlight the toll taken by the constant struggle to make ends meet. Many seem resigned to the idea that their social position in society is unlikely to improve significantly, commonly captured in the remark, “it is what it is:”

We’re not going to get the services that should be there. They don’t care, and they want the people in the ’hood to just kill themselves or just die off so that make room for what they might consider better people, better contributors to society.

I believe if the government really wanted to, they can keep their citizens healthy by providing and not making it so difficult to acquire these healthy choices…. I don’t know what’s going on with the government. If they’re doing this on purpose or not, but food, healthy choices for food shouldn’t be a privilege.

We ain’t going to eliminate it [health disparities]—that’s for one thing—you ain’t going to eliminate none of that. We’re not going to eliminate the health disparity because it is what it is. Now you’re talking economics. Now, you’re getting into the realm of economics. Health disparity is going nowhere. I don’t think disparity is going to go nowhere because it’s a money thing. It’s a money thing to keep people unhealthy.

Examples of intractable obstacles were expressed about the pharmaceutical industry, pesticides in the food supply, pollution and environmental degradation, and the proliferation of carcinogenic materials in consumer products.

#### Tensions between not caring or embracing personal responsibility for health

Widespread cynicism about a better future leaves these men facing a poor choice: falling into a state of not caring about what happens, or assuming personal responsibility for one’s (physical) health. Many participants said that health disparities are the result of feeling that it does not really matter if they live or die, and questioned whether living longer was worth the effort.

Because, like I said, it all comes with the mindset of most of the time we don’t care. We do what we want to do, not caring how it affects our health.

When they are educated on how unhealthy it is, 90% of the time, the response is, “Well, I’m going to die anyway,” so they don’t care.

Black people know the things that it’s not healthy for them, but they do it again. Black people are up against so much competition, so much pressure is being on us today that we know what’s good and we know what’s bad, but we lost confidence in our own selves to be strong as a person and know what’s not good for you and what’s good for you, so we try to do things that’s, we don’t care, we don’t care what we do, not that we don’t know, it’s we just don’t care sometimes.

The perception that their living conditions are unlikely to improve significantly in the foreseeable future leads to a state of apathy, which in turn often gives rise to the desire for whatever ready pleasures might come one’s way. The men expressed the difficult challenges of finding the additional emotional energy necessary to watch one’s weight and diet, as one man put it, “because at the end of the day, you feel sorry for yourself.” Instead, participants spoke of seeking out those immediate pleasures that are available:

For me, I think I’m trying to comply, but you lose yourself in wanting to make yourself feel good by going out to like Golden Corral and eating at an all-you-can-eat buffet and loving the catfish, liking the fried okra, the macaroni and cheese, because ethnically, I grew up with those good-tasting foods, so I’m not going to say no.

From time-to-time when a situation would arise that I would lose control, then I would self-medicate, and I don’t want to, but, yeah, I smoke marijuana, so I can relieve stress that way.

On the other hand, despite high levels of awareness of the constant stress in their lives, many men paradoxically stated that reducing health disparities is strictly a matter of acting with greater personal responsibility.

I feel this way. You’re responsible for your own health. You have a diet, you have health, and you have exercise. You have a variety of things that you can do to better your health. It’s always your choice. There’s consequences of all the choices you make—good or bad.

I’m not using that as a scapegoat, because once I find out that I can do something about it and still choose not to, then that’s on me. When I speak about that—me, being a Black man—once I do know that I can do something about it and I choose not to, there’s a lot of that decision-making that’s going around with most Blacks. They have the opportunity to help themselves health-wise. Sometimes free—sometimes it may cost a little money—whatever it is you’re talking about health, they choose not to do it. The consequences take care of themselves. They have nothing to complain about. This is probably one of the reasons why the rate is so high. The others chose to do something about it.

Summarizing the difficult choice, one participant concluded, “It’s my fault that I don’t do that, but you can’t do these things and stay healthy. It’s all a personal choice.”

## Discussion

Although the identification of different factors in different domains that influence African men’s health is not new, (Griffith, Cornish, Bergner, Bruce, & Beech, 2018; Griffiths, 2012; Watkins, Walker, & Griffith, 2010) the hierarchical ranking of primacy of influence, their synergistic interactions, and the seemingly paradoxical reactions to extant social structural constraints are important new findings to emerge from this study, with significant implications for intervention development.

Based on the findings presented above, it appears that barriers to active participation in the workforce are the most acutely felt driver of middle-aged African-American men’s mental and physical health. The perceptions of our study participants regarding these social structural influences on health are substantiated by empirical measures of levels of unemployment, the lack of progress in closing income gaps, and their combined effect on health.

There are multiple measures of unemployment used in the USA; the most well-known is the Bureau of Labour Statistics “U-3” measure. The U-3 measure is defined as the number of unemployed persons divided by the civilian labour force, but it excludes persons “not in the labor force,” defined as “those who have been out of work for 27 weeks or more, those who are not looking for work” and “institutionalized” persons (in prison or school). The official U-3 unemployment rate in the USA is now 4.3% for the general population, while it is 9.5% for African-American men. Less frequently cited in the press, the “U-6” rate is a wider measure of unemployment, which includes discouraged and “marginally attached” workers (e.g., part-time seasonal workers). According to analyzes by political economist, Nicolas Eberstadt, (Eberstadt, 2016, 2017) when one includes discouraged and institutionalized men, “By 2015, nearly 22% of US men between the ages of 20 and 65 were not engaged in paid work of any kind.” Eberstadt goes on to state that this is an estimate for the population as a whole, noting that rates of joblessness are significantly higher among the less educated, African-Americans, and people with criminal records. If rates of joblessness are compounded by low levels of education, race, and criminal records, and people with these characteristics tend to get concentrated in certain neighbourhoods, then the perceptions of our respondents that most men in their neighbourhoods cannot find work appears well-founded.

Moreover, recent analyzes by Campos (Campos, 2017) show that the percentage gap in average income between non-Hispanic Caucasians and African-Americans has remained virtually unchanged since 1967. Over the last 50 years, African-Americans have continued to bring in roughly half of what non-Hispanic Caucasian workers make, and indeed, the gap has actually worsened in three of five quintiles, while remaining unchanged in a fourth, and narrowing only slightly for those in the second lowest quintile. As these data indicate, there are strong empirical foundations that support the perception that joblessness has a significant impact on physical health; that joblessness and poverty are widespread among African-American men; and that economic conditions for African-American men are not improving.

Krueger (Chira, 2016; Krueger, 2016) has documented gaping differences in health status between men who are working versus men not in the labour force: where 12% of employed men (ages 25–54) have poor or fair health, fully 42% of men not in the labour force have poor/fair health, a more than three-fold difference. The participants’ perception that unemployment was the most significant influence on their health is thus supported by the results of general population surveys (see also, Barr, 2008 (Barr, 2014)). In a more ideal world, we can only speculate that the virulence of racism would abate as economies approached full employment and a decent guaranteed living income.

In addition to the hierarchical ordering of risks, the results of this research extend previous research on the social determinants of health by revealing the direction and types of interactions of different factors across domains. Although presentations of ecological models often make passing reference to potential interactions among different social determinants, these models are commonly depicted in schematic diagrams with arrows linking virtually all the different domains, with little indication of magnitude, primacy or explicit pathways (see, for example [34, 35]). In such frameworks, it is the cumulative total of insults that is considered to result in incremental declines in health.

In contrast, the findings reported here indicate that the different factors are not isolated and independent of one another, but rather, interact synergistically, giving rise to a multiplier effect of one factor setting off another in a growing snowballing effect across domains. As poverty and unemployment, exacerbated by racism, create different problems in the various spheres of daily life, the cascade of effects appears to spread and grow, generating new and additional problems in housing, transportation, food, access to social services and other areas. The cumulative effect results in constant high levels of stress, which reportedly have a powerful effect on these men’s mental health.

The findings presented here must be interpreted cautiously, with the standard limitations that apply to small qualitative studies. Due the small sample size, it is unknown how representative these results may be for the general population of African-American men living in the USA, and hence, the extent to which they may be generalized. Despite these limitations, we feel confident that relieving poverty and unemployment among African-American men is likely to have the most pronounced effect on improving their overall, physical, mental and social health.

In the 60 years since the Selma to Montgomery voting rights marches, the frustrations of continuing high unemployment levels, the unchanging gulf in relative income and wealth, and other significant sources of stress, such as the highly disproportionate mass incarceration rates and the serial slaying of young African-American men by the police, have festered to the point where many poor African-American men have become quite cynical about chances for a better life. As prospects for living the American Dream, of participating in increasing prosperity from one generation to the next, recede into the distant future, the results here reveal contradictory responses to perceived structural barriers. On the one hand, many men have become resigned to not caring about the long-term health consequences of indulging in an unhealthy diet and sedentary lifestyle. On the other hand, with little else they feel they can control, many men assert that any health disparities experienced by their community are due to the failure to accept personal responsibility for health.

Research by Carol Graham (2017) helps to explain the mechanisms by which poverty affects individual well-being, beyond the lack of access to material resources. Graham has found a highly consistent relationship between poverty and measures of stress, insecurity and lack of hope in international research, work that she has recently begun to apply to the USA. In her emerging framework, stress is associated with the constant uncertainty of enduring circumstances beyond one’s control, circumstances where people do not believe that they have the resources to cope with the hardships facing them. Significantly, Graham has found that constant exposure to high levels of stress powerfully shapes people’s attitudes towards the future. Individuals growing up in situations of constant stress tend to focus on more short term outlooks, precisely because, she notes, future prospects appear so uncertain. In contrast, people who grow up in circumstances with less stress have the mental space to think about and make plans for purposefully pursuing future opportunities, especially in terms of attaining rewarding work that can provide a sense of meaningful accomplishment. She terms the different outlooks, respectively, hedonic versus evaluative well-being. When one’s time horizon gets foreshortened by circumstances beyond one’s control, people adopt a hedonic sense of well-being, focusing on the experiences of their daily lives: what can bring them some degree of pleasure and happiness right now. When one can lead a life relatively free from incessant worries and hardships, people adopt a broader evaluative perspective, thinking about what they want to do with their lives in the long term. It is much easier to forgo the immediate pleasures of cheap, fatty foods when one can see more rewarding goals down the road. As one participant commented, “Instead of trying to see our self in a couple years doing good, we worry about right now.”

The results of this research have significant implications for the development of more effective interventions to reduce chronic disease rates in African-American men, and more broadly, health disparities in the general population in international settings. With the recent waves of immigration to Europe, the political situation has become highly volatile, in particular, with respect to the resurgence of nationalism and ethno-centrism, which some have suggested is due, in turn, to the perceived threat to employment and job security. (Molly, 2016; Shiller, 2016) Because the different factors that influence African-American men’s health do not operate independently of one another—where one might assume that each could be tackled separately, solving problems one by one, thereby incrementally improving health—the results presented here provide suggestive evidence that the many separate individual problems cannot be resolved unless or until issues in other domains are addressed as well.

Finally, the recent surge of interest in minority stress models has begun to substantiate that the impact of the macro-level social determinants on health appears to be mediated at the individual level by stress, objectively confirmed in physiological measures of cortisol and allostatic load. (Geronimus, Hicken, Keene, & Bound, 2006) As Graham’s research cited above suggests, exposure to chronic stress molds attitudes towards the future and perceived possibilities for pursuing fulfilling long-term lifeplans. Such findings have significant implications for the oft-stated goal of developing culturally competent interventions. Although we found that our participants often have contradictory responses to their observed chances of finding decent work, the aim of changing cultural norms by tailoring messages in cultural compatible terms fails to address the underlying causes that foreshorten hope into the pursuit of momentary hedonic rewards. Moreover, on an ethical level, Shelby (Shelby, 2016, 2015) has mounted trenchant critiques of the ostensible ethical justification for developing culturally competent interventions designed to change the attitudes, norms and behaviours of marginalized, low-income African-American communities by well-intended, liberal health and social service agency professionals.

In conclusion, just as many people assert that health disparities result from the failure to accept personal responsibility, an analogous line of thinking is evident with respect to the causes of poverty. Historically, the dominant majority of Americans have accepted high levels of inequality because they have believed that income and wealth are directly related to level of individual effort: the rich are rich because they work hard, and the poor are poor because they do not. People think that the system is fair to the extent that they believe that upward mobility is within anyone’s reach, if only one works hard enough for it. Until quite recently, the myth of Horatio Alger has survived largely intact because there were large groups of people that the dominant majority did not consider part of the larger social collective. Whether consciously or unconsciously, the plight of the poor and minorities could be ignored, pass unrecognized and unappreciated by the majority in service to the ideal that America is a just society: Americans like to believe that everyone not only can but is now getting ahead. (Kraus, Rucker, & Richeson, 2017) Against this idealistic illusion, by conservative estimates, one-third of African-American men are out of work. To relieve the stress associated with significant health disparities driven by social structural policy failings will require an unprecedented effort to forge bonds of social solidarity (Dawson & Jennings, 2012; Dawson & Verweij, 2012) and embrace a new larger collective identity, one where citizens accept collective responsibility for the mental and physical health and well-being of all.
